# Evolving concepts for safer ventilation

**DOI:** 10.1186/s13054-019-2406-9

**Published:** 2019-06-14

**Authors:** John J. Marini

**Affiliations:** University of Minnesota, Regions Hospital MS 11203B, 640 Jackson St, St. Paul, MN 55101 USA

**Keywords:** ARDS, Lung injury, Mechanical ventilator, Mechanical power, Driving pressure, Lung stress and strain

## Abstract

Our current understanding of protective measures for avoiding ventilator-induced lung injury (VILI) has evolved from targeting low tidal volumes to lowering plateau and driving pressure. Even when pressures across the lung are reliably estimated, however, pressures alone cannot accurately gauge the injury risk; apart from flow rate, inspired oxygen fraction, and currently unmeasurable features of the mechanical microenvironment such as geometry, structural fragility, and vascular perfusion, the frequency with which high-risk tidal cycles are applied must help determine the intensity of potentially damaging energy application. Recognition of a strain threshold for damage by transpulmonary pressure, coupled with considerations of total energy load and strain intensity, has helped shape the unifying concept of VILI generation dependent upon the power transferred from the ventilator to the injured lungs. Currently, under-recognized contributors to the injury process must be addressed to minimize the risk imposed by ventilatory support.

## Introduction

**Fig. 1:**
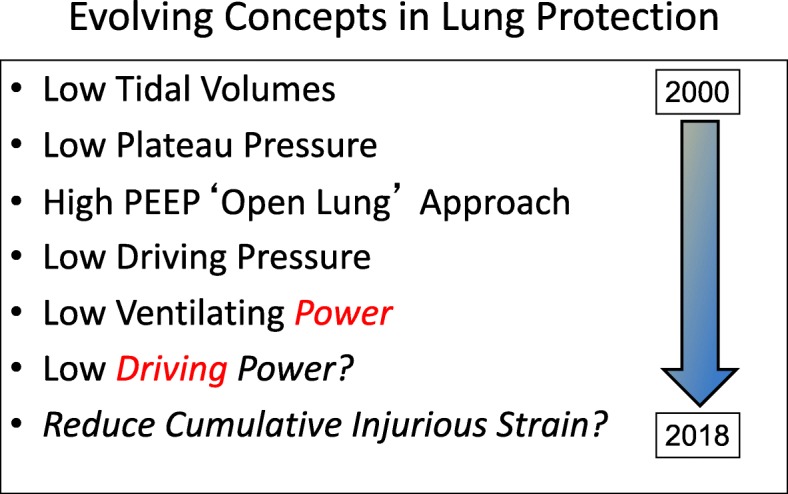
Timeline of advancing knowledge regarding VILI causation

Since the year 2000, our understanding of protective measures for avoiding ventilator-induced lung injury (VILI) has evolved from targeting low tidal volumes [1] to lower plateau pressure, more “open” lung [2, 3], lower driving pressure [4], lower ventilating power [5], lower driving power [6], and reduced frequency of injurious strain cycles [6, 7] (Fig. 1). Tacitly underlying all forms of ventilation-inflicted injury, however, is the understanding that energy and power are required to produce it. Static pressures at the extremes of the tidal cycle (plateau and PEEP) represent forces that are counterbalanced, do not expend energy, and cannot cause injury—only transitions between static pressures can do that.

In turn, lung stress and the resulting strain it produces relate to the trans-alveolar (transpulmonary) pressure applied during the tidal breath [8]. Clinicians have been slow to acknowledge that airway pressures provide incomplete information concerning the lung and to adopt the use of the esophageal balloon catheter to estimate the pressure that surrounds it. At the bedside, most practitioners continue to rely on measurements of airway pressure and flow. These serve well to track the energy load imposed upon the series-coupled (lungs and chest wall) of the respiratory system by the ventilator during passive inflation. In the injured lung, actual strains are heightened at junctions of closed and open lung units, and these stress-focusing points (as well as mechanical heterogeneity) are less in the prone position. In any position, single breathing cycles clearly do not inflict detectable injury; multiple trans-alveolar pressure cycles that exceed safe limits are required. To comprehend the root mechanical cause of ventilator-induced lung injury, a basic knowledge of mechanical energy transfer is required, as no damage results at rest from static pressure alone.

## Basic concepts of energy and power

Mechanical work performed upon an object, and consequently the energy it requires, is defined as the unbalanced force applied times distance moved by that force. Power is the rate at which work is performed per unit time and can be measured on any time scale—e.g., minutes, seconds, or within a portion of the breath itself. One key tenet of physics is that energy can neither be created nor destroyed but only transformed among its varied forms. There are multiple types of energy (radiant, thermal, nuclear, etc.), but during ventilation, mechanical energy and work are the relevant domain. Here, mechanical energy phasically transitions between potential energy and kinetic energy. The latter is spent either in generating heat against resistance or in producing deformation and damage. During the tidal breath, potential energy is stored in the elastic tissues of the lung and chest wall during the inflation phase. This potential energy is expended during expiration in overcoming the resistances of the tissues, airways, and artificial apparatus (endotracheal tubes, external connecting circuitry, and expiratory valve). The work performed during inflation on the lung or passive respiratory system is the product of pressure and volume [9]. Pressure is force per unit area, and volume is the product of the area and length. Multiplying pressure by volume therefore yields the force-length value that defines the mechanical work performed. When pressure is quantified in centimeters of water and volume in liters, the energy unit is the joule (J). The watt is the power unit—energy expended per unit time—indicating expenditure of 1 J/s.

Classical physiology describes the equation of motion for the respiratory system as the sum of flow resistive pressures and elastic pressures [10]. Thus, total inflation pressure is the sum of flow times resistance plus the integral of flow (*F*) divided by compliance (*C*) (The latter quotient is known as the “driving pressure.”), plus the total end-expiratory pressure (PEEPtot = applied PEEP plus auto-PEEP) from which inflation began. At end inspiration, therefore:

$$ \mathrm{Ptot}=\mathrm{FR}+{V}_{\mathrm{T}}/C+\mathrm{PEEPtot} $$

At the end of one inflation cycle, the input energy has been the product of pressure and the corresponding tidal volume:

$$ \mathrm{Energy}={V}_{\mathrm{T}}\times \mathrm{Ptot}={V}_{\mathrm{T}}\times \left[\mathrm{FR}+{V}_{\mathrm{T}}/2C+\mathrm{PEEPtot}\right] $$

**Fig. 2:**
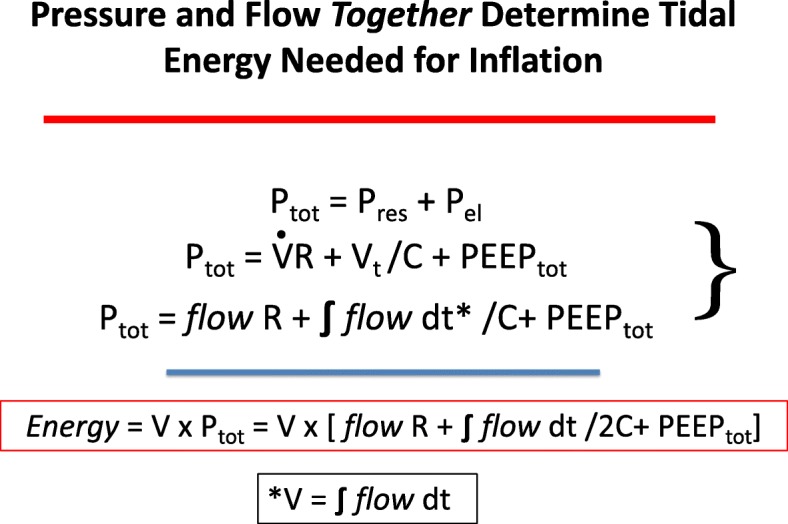
Flow, volume (*V*), and pressure (*P*) determinants of the total inflation pressure and energy needed for tidal inflation [*V* overdot = inspiratory flow]

**Fig. 3:**
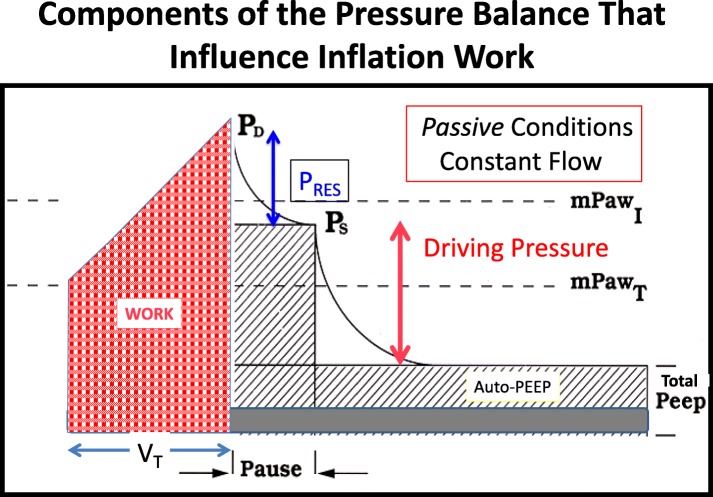
Components of the total inflation pressure that determine inspiratory work during constant inspiratory flow achieved under passive conditions. mPaw_I_ mean inspiratory pressure, mPaw_T_ mean airway pressure, *P*_D_ peak dynamic pressure, *P*_S_ static end-inspiratory (plateau) pressure, *P*_RES_ flow resistive pressure

In this expression, *V*_T_ must be divided by 2 to appropriately reflect the average tidal elastic (driving) pressure. Inflation work during constant inspiratory flow can be considered as the sum of the three energy components relating to flow resistance, driving pressure, and total PEEP, as illustrated in Fig. 2. It should be noted that under constant flow conditions, time and volume are linear analogues and that the pressure-time (volume) area graphically illustrates the product that defines work (Fig. 3) [11].

## Potential damage from the tidal energy components

Flow resistance work is performed in pushing gas to the alveolar level. During that process, frictional losses occur not only along the airway epithelium but also within the parenchyma itself [12]. Indeed, in setting of ARDS, such tissue losses are considerably higher than they would be during health. Therefore, when considering the potential for the flow rate to contribute to tissue damage, such frictional parenchymal losses of inflation energy cannot be ignored. Especially relevant to the frictional stresses imposed by flow is the property of viscoelastance [12]. As the lung expands, some interlaced components of the interstitium that separate individual alveoli lag behind others, heightening the imposed stress upon the elements that separate them. Such stresses and strains are increased by higher rates of flow [13].

The second component of inflation work is that which relates to the driving pressure, i.e., the quotient of tidal volume and respiratory system compliance. During constant flow, this triangular pressure-volume product is maximized at tidal volume, so that this quantity must be divided by a factor of 2 to obtain the average that pertains to the entire inflation. The effects of this energy component are concentrated by the low capacity of the baby lung to accept it [6]. Yet, although driving power may be central to the mechanical forces that cause lung injury, it does not act alone. Not only is the rate at which stress applied important to the consequences of strain, but also the level of tissue tension that existed prior to its application strongly influences the tissue’s proximity to the strain threshold for damage and therefore the impact of a given amount of driving power. The latter is conditioned by the level of end-expiratory pressure from which the inflation begins, i.e., total PEEP [7].

Even though PEEP is not traditionally considered a primary component of the work of breathing because it does not incorporate flow and tissue movement, it does set the platform of pressure upon which driving pressure operates. To increment pressure by a given amount, a greater force is necessary when starting from a higher level of pressure. Therefore, when starting the breath from an already raised pressure baseline, more inflation energy is required than without PEEP. It stands to reason that when PEEP is applied, the higher pressure required to achieve a given tidal volume may exceed the pressure threshold for inciting damage [14, 15].

## Driving pressure importance and limitations

It follows from this analysis that a given numerical value for driving pressure—even one based on transpulmonary pressure—should not be considered safe or dangerous without specifying other conditions, such as the starting pressure (PEEPtot) and potential for tidal recruitment and atelectrauma [16]. Whereas PEEP applied to a recruitable lung may reduce the number of vulnerable lung units as the associated higher pressures recruit them, those lung units that remain closed or unstable are put under increased stress and strain [17]. Low compliance alveolar units experience less distention and potentially damaging strain for a given driving pressure. As PEEP increases, the net benefit or hazard may remain unchanged, rise, or fall.

Driving pressure, the difference between two static airway pressure variables (Pplat and PEEPtot) for the individual tidal cycle, cannot be considered as the final and exclusive key to VILI prediction or moderation. Clearly, it seems to be a better predictor of some key outcomes than either of its defining elements [4]. But it holds the potential to underestimate the ventilation risk, depending on the number and intensity of stress-focusing points, effects of viscoelastance (as reflected by the difference between the end-inspiratory pressures at zero flow [P1] and the plateau [P2]), or surreptitious and undetected inspiratory muscle tone. Conversely, driving pressures derived from airway pressure recordings alone may overestimate the risk to a lung surrounded by a stiff chest wall. Use of transpulmonary pressure helps circumvent both the muscle activity/tone and chest wall stiffness contributions to raw airway pressure.

Even when the pressures across the lung are reliably estimated, however, pressures alone cannot accurately gauge the injury risk; the frequency with which high-risk cycles are applied determines the intensity of potentially damaging energy application. This consideration of energy load evolved into the unifying concept of VILI generation by the power transferred from the ventilator to the lungs [5, 15]. Debate continues as to whether the entirety of the inflation power components—resistive, tidal elastic, and PEEP—or simply the driving power (with flow resistive and PEEP-related energy portions sharply discounted) is principally responsible for VILI causation [6, 7]. Nonetheless, flow rate and PEEP are theoretically influential for reasons already mentioned, and both have been shown experimentally to be potentially important conditioners of the driving pressure [18, 19].

Another key addition to our understanding is that the experimental thresholds for lung injury to occur are both relatively high and sharp in the normal lungs of both small [20] and large [5, 21] anesthetized animals. The injury threshold is likely to be considerably lower in the mechanically heterogeneous microenvironments of already injured lungs. It follows that because rather high numerical values for power can be achieved without crossing the tidal threshold of damaging strain [22], neither power nor driving power themselves is an unassailable candidate for the key to VILI. If it were, then high-frequency ventilation at virtually all mean airway pressures would prove rapidly intolerable, as these modes deliver extraordinarily high levels of power and driving power. With only rare exception [23], such intolerance has not proven to be the case.

## Can unaccounted “absorbed” power account for injury?

While very attractive, the power-injury hypothesis seems incomplete for another important reason: much of the elastic energy stored in the lung at end inspiration in its potential form can be accounted for as the energy needed for overcoming expiratory resistance. Considering only the component related to PEEP, for example, the amount of kinetic energy released to the atmosphere during lung deflation is the product of the pressure across the valve (PEEP) and the integral of flow across it—i.e., the tidal volume times frequency, or minute ventilation [24]. The driving pressure in excess of PEEP is largely spent during exhalation in overcoming expiratory resistance across the airways, endotracheal tube, and conducting circuitry of the external ventilator circuit. Because energy must be accounted for in “zero-sum” fashion among its three major forms (potential, kinetic-resistive/heat, and kinetic-damage), our ability to account for most stored end-inspiratory elastic energy in dissipation against resistance leaves relatively little residual to directly inflict mechanical injury. Perhaps signaling by stretch is a more attractive explanation? We cannot be sure.

A component of the energy supplied during the tidal cycle does remain unaccounted for, and this “hysteresis-related” pressure-volume loop area would seem the most likely region on which to focus attention (Fig. 2). This “absorbed” energy during the breath might be expended in first expanding and then re-folding the lung parenchyma into its pre-inflation state, in overcoming parenchymal viscoelastance during the rapid phases of inflation and early deflation, or perhaps in causing direct mechanical damage to structural microelements. While strong forces may tear the alveolar-capillary barrier in demonstrable fashion, such small amounts of hysteresis energy would seem unlikely to do so. It is fair to question whether such small amounts of energy and power could cause the VILI that we eventually see [7].

## Amplifiers of “unaccounted” energy

Tissue damaging events occur at the microscopic level, and any numerical value of energy or power must be considered in this context. “Lost” energy which focused on a very small and vulnerable region would have accentuated impact. The interstitial compartment is made up of interdependent microfibrils that do not have the resilience or robust elasticity of strong collagen. Three relatively obvious mechanisms of force amplification may affect this compartment, setting into motion overt rupture of its microelements or exerting sufficient interstitial tension to initiate inflammatory signaling within the cells that define it. Those three mechanisms are geometric stress focusing, viscoelastic drag, and progressive drop-out of the stress-bearing microelements [7].

Geometric stress focusing occurs at the boundaries of tissues with different mechanical properties due to asymmetry. Such mechanisms are well known to materials engineers and were borrowed for application to the biological setting of the lung nearly a half century ago by Mead, Takishima, and Leith [25]. The amplification coefficient is dependent on the applied pressure but may theoretically rise to 2–3 at the upper boundary of plateau pressures in clinical use. The second mechanism accentuating the forces applied to the interstitial microelements is a viscoelastic drag, an amplifier that varies with the rate of flow [12]. It should be emphasized that the effective flow experienced by the aerated portion of the “baby lung” may be considerably greater than when more lung units and accompanying conducting airways were available to accept the tidal volume [26]. Furthermore, because injured, mechanically heterogeneous tissues experience greater reluctance to expand and deflate in unison, interstitial tensions imposed on the microelements increase, especially when volume changes occur quickly.

The third important force amplifier is sequential drop-out among parallel load-bearing microstructures of the interstitial space [27]. A load of “*x*” amount applied to fewer supporting elements will exert a greater effect upon each of them than would be experienced by more of them sharing the total burden. As the weaker microelements break, their individual supporting contributions are lost, transferring the entire stress load to those that remain intact. This is an accelerating process that may help to explain why damage may proceed in exponential fashion once some critical level of tension per microelement has been crossed. Acting in conjunction with viscoelastic drag and geometric stress focusing, such amplification makes plausible the assignment of lost hysteretic energy to VILI causation.

## Potential for damage during tidal exhalation

Until quite recently, the entire focus on VILI and its avoidance was directed to the inflation half cycle, with deflation considered to be an entirely passive process. While such a concept may be appropriate for the healthy lung, this exclusivity may not be warranted for the mechanically heterogeneous environment of the already injured one. Energy is required to re-establish the lung’s end-expiratory resting state, and the stress amplifying mechanisms just described apply equally well to the first phase of lung deflation. The diaphragm relaxes only gradually and partially during deflation, in part to avoid end-expiratory atelectasis [28], but perhaps also to reduce the abruptness and unevenness of deflation. The latter two processes augment tissue stresses during the initial portion of tidal exhalation. Intriguing experiments performed by Schumann and colleagues have repeatedly demonstrated that flow limitation during early expiration (“FLEX”) without generating auto-PEEP helps preserve lung performance and limits the evidence of VILI [29, 30]. If so, such modifications may be needed in settings where the diaphragm cannot act normally as an effective flow governor (e.g., pharmacoparalysis [31]).

## Non-mechanical co-factors of VILI causation

In any discussion of evolving concepts of VILI, the *co-factors* that condition the effect of the mechanical stresses should not be ignored. Unnecessary increases of inspired oxygen are likely co-contributory [32]. But perhaps the most important among these co-factors are the vascular flows and pressures perfusing the damaged lung [33]. An increase of cardiac output necessitates higher mean pulmonary artery pressure as well as widening of the vascular pressure gradient across the alveoli. Raising mean pulmonary arterial pressure encourages edema formation within the high permeability lung, and in turn, this tissue liquid increases the tendency for mechanical heterogeneity [34]. An increased gradient of microvascular pressure implies greater energy dissipation across the endothelium, another form of “ergotrauma.” In the ventilated lung of ARDS, some regions undoubtedly cycle repeatedly between West “zone 2” on inflation and “zone 3” on deflation. The resulting vascular surge of energy flowing across the injured endothelium has the potential to accentuate damage, providing another argument for avoiding unnecessarily high swings of alveolar pressure that accompany forceful spontaneous efforts, inappropriately large tidal volumes and high plateau pressures [35].

## Summary

Knowledge regarding VILI has progressed relatively rapidly in recent years from a restricted view of volumes and pressures associated with the individual tidal cycle to considerations of energy load and power that emphasize the intensity of tidal stress and strain application. At the present time, the process of VILI is believed to be a rather complex one, whose multiple causes involve multiple mechanistic threads and both the inflation and deflation phases of ventilation. Funneled into a single “umbrella” concept, investigators are now targeting avoidance of excessive power exposure coupled to repeated imposition of breaths that encourage excessive tissue strain. Limiting driving pressure, flow rate, PEEP, and frequency while avoiding widespread collapse is seen as essential. Attention to reducing mechanical heterogeneity by prone positioning [36], lowering the inspired oxygen fraction (FiO2), and diminishing demands for ventilation and cardiac output are other keys to what is now viewed as a rational lung-protective approach.

## Abbreviations

*C*: Compliance
*F*: Flow
FiO2: Inspired oxygen fraction
FLEX: Flow limitation on expiration
P1: End-inspiratory pressure at first point of zero flow after airway occlusion
P2: Plateau airway pressure after an imposed pause at end inspiration
PEEP: Positive end-expiratory airway pressure
PEEPtot: Total positive end-expiratory alveolar pressure
*R*: Resistance
